# Per-partnership transmission probabilities for *Chlamydia trachomatis* infection: evidence synthesis of population-based survey data

**DOI:** 10.1093/ije/dyaa202

**Published:** 2020-12-08

**Authors:** Joanna Lewis, Peter J White, Malcolm J Price

**Affiliations:** 1 MRC Centre for Global Infectious Disease Analysis and National Institute for Health Research (NIHR) Health Protection Research Unit in Modelling and Health Economics, Imperial College London School of Public Health, London, UK; 2 Centre for Applied Statistics Courses, UCL Great Ormond Street Institute of Child Health, University College London, London, UK; 3 Modelling and Economics Unit, National Infection Service, Public Health England, London, UK; 4 Institute of Applied Health Research, University of Birmingham, Birmingham, UK; 5 NIHR Birmingham Biomedical Research Centre, University Hospitals Birmingham NHS Foundation Trust and University of Birmingham, Birmingham, UK

**Keywords:** Chlamydia, transmission, mathematical model, Bayesian statistics, evidence synthesis, population-based survey

## Abstract

**Background:**

Chlamydia is the most commonly diagnosed sexually transmitted infection worldwide. Mathematical models used to plan and assess control measures rely on accurate estimates of chlamydia’s natural history, including the probability of transmission within a partnership. Several methods for estimating transmission probability have been proposed, but all have limitations.

**Methods:**

We have developed a new model for estimating per-partnership chlamydia transmission probabilities from infected to uninfected individuals, using data from population-based surveys. We used data on sexual behaviour and prevalent chlamydia infection from the second UK National Study of Sexual Attitudes and Lifestyles (Natsal-2) and the US National Health and Nutrition Examination Surveys 2009–2014 (NHANES) for Bayesian inference of average transmission probabilities, across all new heterosexual partnerships reported. Posterior distributions were estimated by Markov chain Monte Carlo sampling using the Stan software.

**Results:**

Posterior median male-to-female transmission probabilities per partnership were 32.1% [95% credible interval (CrI) 18.4–55.9%] (Natsal-2) and 34.9% (95%CrI 22.6–54.9%) (NHANES). Female-to-male transmission probabilities were 21.4% (95%CrI 5.1–67.0%) (Natsal-2) and 4.6% (95%CrI 1.0–13.1%) (NHANES). Posterior predictive checks indicated a well-specified model, although there was some discrepancy between reported and predicted numbers of partners, especially in women.

**Conclusions:**

The model provides statistically rigorous estimates of per-partnership transmission probability, with associated uncertainty, which is crucial for modelling and understanding chlamydia epidemiology and control. Our estimates incorporate data from several sources, including population-based surveys, and use information contained in the correlation between number of partners and the probability of chlamydia infection. The evidence synthesis approach means that it is easy to include further data as it becomes available.

**Key Messages**

- Estimates for parameters like transmission probability are important for building models of sexually-transmitted diseases that can be used to understand their epidemiology and plan and assess control interventions.
- Average per-partnership (rather than per-sex-act) transmission probability is a particularly useful parameter because there are more and better data on numbers of partnerships than numbers of sex acts.
- We have developed a new method for estimating per-partnership chlamydia transmission probability, using data from population-level studies. We used a Bayesian approach to provide a probability distribution representing the estimate and associated uncertainty.
- We applied our method to the Second National Study of Sexual Attitudes and Lifestyles (Natsal-2) from the UK and National Health and Nutrition Examination Surveys (NHANES) from the USA.

## Introduction

Chlamydia is the most commonly diagnosed sexually transmitted infection worldwide. In 2018 there were 1382 and 3694 chlamydia diagnoses per 100 000 15–24-year-old US men and women, respectively,^1^ and 1342 and 2637 in England.^2^ There is marked geographic variation in chlamydia burden,^3^ and the effectiveness of widespread testing and/or screening in chlamydia control remains uncertain,^4^^,^^5^ but the need for cost-effective control measures becomes ever-clearer as evidence for the link to pelvic inflammatory disease (PID) is strengthened^6^ yet resources for sexual health services are reduced.

Mathematical models are important tools for assessing and predicting the effectiveness and cost-effectiveness of chlamydia control policies. Numerous models have been developed for these purposes^7^ but a comparison of three individual-based models found they produced very different results.^8^ A key parameter in any transmission-dynamic model is the transmission probability per infectious contact, where a ‘contact’ may be defined either as a partnership or as a sex act. Transmission probability has to be estimated indirectly, as it would be unethical to conduct a study measuring it directly, and is subject to significant uncertainty. Modelling studies have used values ranging from 0.0375 to 0.154 per sex act; sometimes assuming equal male-to-female and female-to-male transmission rates, and sometimes allowing for a higher risk in the male-to-female direction.^7^

Transmission probability estimates can be based on cross-sectional concordance studies of sexual partnerships. For example, Katz used data from a US clinic to estimate the proportion of heterosexual couples forming in which the man only, the woman only, neither partner, or both are infected.^9^ Using the observed proportion of couples in each state, he estimated the male-to-female and female-to-male transmission probabilities over the time between partnership formation and observation.^9^ However, concordance was observed before the partnership ended, and so the estimated transmission probabilities represented only transmission before observation – not the full per-partnership probability. Furthermore, these estimates do not allow for recovery and/or re-infection within a partnership. Althaus *et al*. proposed an alternative model based on differential equations which explicitly incorporated partnership formation and breakage, occurring with constant hazards.^10^ The analysis is informative but the estimates it provides depend on values assumed for other parameters in the model, some of which are not well-defined; in particular, the duration of infection and the number of partnerships in the last 6 months. Finally, transmission probabilities can be estimated by calibrating a transmission model to population prevalence data.^11^ With this approach, the values estimated depend on the data to which the model is calibrated, the values of other parameters, and the structural assumptions in the model.

In this paper we develop a different approach. We calculate average per-partnership chlamydia transmission probabilities from an infected man to an uninfected woman and from an infected woman to an uninfected man, using data from two population-based surveys: the 1999–2001 UK National Survey of Sexual Attitudes and Lifestyles (Natsal-2)^12^ and the 2009–2014 US National Health and Nutrition Examination Surveys (NHANES),^13^ synthesized with information on the clearance rate of untreated chlamydia infections. The method avoids many of the assumptions that are required for estimation within a dynamic model, and its reliance on other unknown quantities is minimal and well-described. Furthermore, because estimates are based on data from population-based surveys, the results are directly applicable to the general population. The methods could also be applied to other sexually transmitted infections with a susceptible–infected–susceptible (SIS) model of natural history.

## Methods

The aim of the study was to provide a mathematical and statistical model that can be used to infer per-partnership transmission probability from survey data. We present an overview of our methods; further details are in the Supplementary Information, available as Supplementary data at *IJE* online.

### Mathematical model

**Figure 1:**
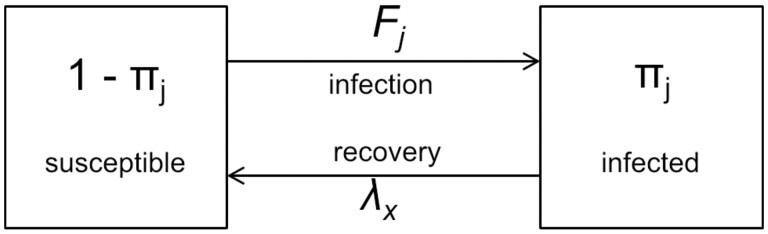
SIS model of chlamydia infection and recovery for individual *j*, of sex *x*. $\pi_{j}$ is the probability of being infected with chlamydia and $1-\pi_{j}$ is the probability of being susceptible. $F_{j}$ is the force of infection and $\lambda_{x}$ is the recovery rate

We used an SIS model of infection and recovery (Figure 1). Our model considers asymptomatic infections; symptomatic infections prompt treatment seeking and are therefore short-lived and unlikely to cause onward infection or to be detected in population-based surveys.

Let each individual *j*, of sex *x*, experience a force of infection *F_j_*. This force of infection (accounting for heterosexual transmission only) is the rate at which an individual makes contacts with infected members of the opposite sex, $\chi_{xj}$, multiplied by the per-contact transmission probability, $\rho_{x'\to x}$:

$$F_{j}=\chi_{xj}\rho_{x'\to x}.$$

(*x′* denotes the opposite sex to *x*.)

Individuals’ recovery rate is $\lambda_{x}$. The probability that individual *j* is infected at a given moment is $\pi_{j}$. At steady state, the number of new infections per unit time ($F_{j}\left(1-\pi_{j}\right)$) equals the number of recoveries ($\lambda_{x}\pi_{j}$,):

$$F_{j}\left(1-\pi_{j}\right)=\chi_{xj}\rho_{x'\to x}\left(1-\pi_{j}\right)=\lambda_{x}\pi_{j}$$

Hence,

$$\rho_{x'\to x}=\frac{\pi_{j}}{1-\pi_{j}}\times \frac{\lambda_{x}}{\chi_{xj}}$$

### Data

We inferred parameter values in the model by synthesizing data from several sources.

#### Clearance of untreated chlamydia infection

Data informing the clearance rate of untreated infections came from studies in the literature synthesized in previous analyses.^14^^,^^15^ Further details are provided in the original papers.^14^^,^^15^

#### Numbers of partners

We used data on sexual behaviour and chlamydia infection from two population-based studies: Natsal-2,^16^ and the three NHANES conducted biennially between 2009 and 2014.^17^ We combined data from three NHANES to achieve a larger sample size than would be possible using only one.^17^*

In Natsal-2, participants reported on their number of new opposite-sex partners in the last year, and this information was used to inform a probability distribution for the number of new partners in the last year.

In NHANES, participants were asked their number of partners, and whether they had had any new partners, in the last 12 months. We used these two questions to provide a proxy for the number of new partners in the last year. Where respondents reported no new partners in the last year, we took the number of new partners to be zero; where they reported one partner and a new partner, we took the number of new partners to be one; otherwise, we assumed that all but one of their total reported partners was new. This approach is similar to the use elsewhere of ‘shifted negative binomial’ distributions for modelling partner numbers.^18^

#### Infection status

The publicly-available data from both Natsal-2 and NHANES also includes chlamydia infection status, diagnosed using nucleic acid amplification tests (NAATs) on urine samples. Natsal-2 participants were eligible for a urine sample if they were aged 18–44 years and had ever had sex, and a randomly-selected half of those eligible were invited to provide samples. All NHANES participants aged 14–39 years were invited to provide a sample for testing, but the publicly-available data excludes 14–17-year-olds.

Numbers of partnerships reported by susceptible and infected men and women in Natsal-2 and NHANES are provided in Supplementary Tables 1 and 2, available as Supplementary data at *IJE* online.

### Statistical model

We conducted a Bayesian evidence synthesis, using data from the sources described, to construct a likelihood. Survey weights were incorporated by multiplying the relevant component of the log-likelihood by the weight. The likelihood was combined with appropriate priors to provide a joint posterior for the model parameters.

#### Clearance of untreated infections

The statistical model used for the clearance rates of untreated chlamydia infection is described elsewhere.^14^ The model involves two courses for infection: fast- or slow-clearing. A proportion *p* of incident infections clear fast, and the remainder, 1 – *p*, clear slowly. Some of the data on chlamydia clearance came from studies using culture diagnosis methods, and the model accounts for this using a sensitivity parameter for culture diagnosis in people with a previous positive culture for that infection, ψ. In this analysis we assumed that only the slow-clearing infections last long enough to be detected in population-based studies. The clearance rate (denoted $\lambda_{x}$ above) is therefore equal to the slow clearance rate in the clearance model, and the transmission probability we estimated is the probability that an infection is transmitted and then follows the slow-clearing course.

#### Partnership dynamics

We used negative binomial distributions to model the estimated numbers of new partners reported in the last year by men and women. A negative binomial distribution with size α and mean μ can arise as a mixture of Poisson distributions, where the mixing distribution for the Poisson rate is a Gamma distribution with shape α and rate $\frac{\mu }{\alpha }$.^19^ In our model, the shape and rate depend on the sex of the individual, but are constrained so that the expected number of partnerships per man must equal the expected number of partnerships per woman.

#### Prevalence

We used our model to calculate the probability $\pi_{j}\;$of each individual *j* being infected, given the number of partners they reported. The infection status of *j* has a Bernoulli distribution with parameter $\pi_{j}$:

$$P\left(\delta_{j}{|\pi }_{j}\right)=P_{\mathrm{Bernoulli}}\left(\delta_{j}|\pi_{j}\right)=\left\{\begin{array}{cc} \pi_{j} & \delta_{j}=1\\ 1-\pi_{j} & \delta_{j}=0 \end{array}\right.$$

where

$$\delta_{j}=\left\{\begin{array}{cc} 1 & \text{if}\;j\;\text{is}\;\text{infected}\\ 0 & \text{if}\;j\;\text{is}\;\text{uninfected} \end{array}\right.$$

#### Full likelihood

The log-likelihood of the data is given by:

$$L=L_{\mathrm{turnover}}+L_{\mathrm{clearance}}+L_{\mathrm{infection}}$$

where:

- $L_{\mathrm{turnover}}$ is the log-likelihood associated with partnership turnover (negative binomial distribution);
- $L_{\mathrm{clearance}}$ is the log-likelihood associated with clearance, and
- $L_{\mathrm{infection}}$ is the log-likelihood associated with the infection status of each participant at the time of testing in the survey (Bernoulli distribution).

### Inference and estimation

#### Priors

We used uninformative priors for all parameters except the sensitivity of chlamydia diagnosis by culture, which enters the model for chlamydia clearance. This had a $\psi \;∼\;\text{Beta}\left(78,8\right)$ prior, based on studies comparing the performance of culture diagnosis and NAATs.^14^

#### Bayesian methods and sampling of posterior distribution

Estimation was carried out by sampling from the posterior using a Markov chain Monte Carlo (MCMC) algorithm implemented in the Stan software,^20^ within the R environment.^21^ The data, Stan model file and R scripts used for handling input and results are all available online at https://github.com/joanna-lewis/ct_transmission_probs. We ran four chains for 2000 iterations each, discarding the first 1000 ‘warmup’ iterations of each chain. Posterior predictive checks were carried out, comparing simulated and observed partner number distributions, and prevalence in men and women reporting different numbers of partners. We also used prior distributions for the proportion of infections leading to symptoms for men and women to simulate the annual number of symptomatic infections that would have occurred under the parameter values inferred (see Supplementary Information, available as Supplementary data at *IJE* online).

### Sensitivity analysis

We conducted three sensitivity analyses to investigate different aspects of our model, which are described in detail in Supplementary Information, available as Supplementary data at *IJE* online. First, we relaxed the assumption of equal average numbers of partnerships in men and women. Secondly, we constructed a model in which individuals only form partnerships with members of the opposite sex reporting the same number of partnerships. This tests two aspects of the model: (i) by imposing totally assortative mixing by number of partners, it tests the effect of assuming that partners are chosen at random from all those available; and (ii) by allowing for differing force of infection in individuals reporting different numbers of partners, it tests the effect of using a single average transmission probability across all partnerships. Finally, we used data from Natsal-2 to investigate the effect of studying the number of partnerships without a condom, rather than total partnership numbers.

## Results

For all parameters split $\hat{R}$ statistics for the MCMC sampling were between 0.9990 and 1.0032, indicating good convergence, and the effective sample size was >0.4 per transition of the Markov chain. No transitions ended with a divergence.

In Natsal-2 the mean number of new partners per year was inferred as 0.59 [95% credible interval (CrI) 0.54–0.65]. Overall chlamydia prevalence was 2.1% (95%CrI 1.6–2.8%) in men and 2.0% (95%CrI 1.4–2.8%) in women, compared with survey-based estimates of 2.4% (95%CI 1.5–3.6%) and 1.5% (95%CI 1.0–2.1%). In NHANES the mean number of new partners inferred was 0.92 (95%CrI 0.85–1.00). Prevalence was 1.7% (95%CrI 1.3–2.3%) in men and 3.7% (95%CrI 2.8–4.6%) in women, compared with survey-based estimates of 1.9% (95%CI 1.3–2.6%) and 2.3% (95%CI 1.7–3.0%).

**Figure 2:**
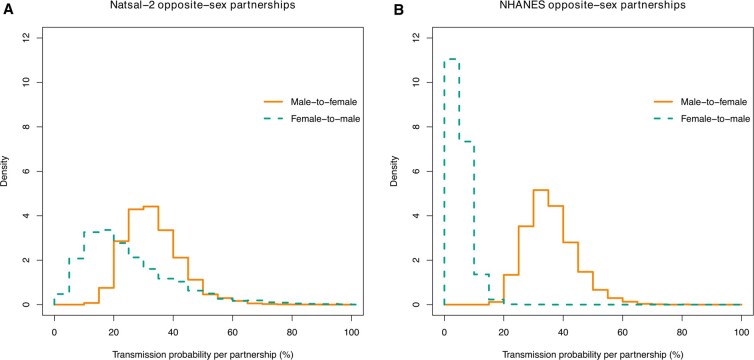
Posterior distributions for the per-partnership probability of chlamydia transmission, derived using number of new partners reported in (**A**) The second National Study of Sexual Attitudes and Lifestyles (Natsal-2), and (**B**) the National Health and Nutrition Examination Surveys (NHANES) 2009–2014 (all studies combined). The yellow line in each figure represents male-to-female transmission probability and the green line female-to-male

Figure 2 shows posterior distributions for the per-partnership transmission probabilities, derived using Natsal-2 and NHANES. Using Natsal-2, the posterior median transmission probabilities were 32.1% (95%CrI 18.4–55.9%) (male-to-female) and 21.4% (95%CrI 5.1–67.0%) (female-to-male). Using NHANES, they were 34.9% (95%CrI 22.6–54.9%) (male-to-female) and 4.6% (95%CrI 1.0–13.1%) (female-to-male). The posterior distributions for all parameters are summarized in Supplementary Table 4, available as Supplementary data at *IJE* online.

Posterior predictions for the partner number distributions generally agreed with data but there was some discrepancy, especially in women (Supplementary Figure 2, available as Supplementary data at *IJE* online). Predicted numbers of infections, by reported numbers of partners, agreed well with observations in both sexes, for both studies (Supplementary Figure 3, available as Supplementary data at *IJE* online).

For Natsal-2 we simulated median (2.5th–97.5th centile) 109 000 (25 000–327 000) symptomatic cases in men; the number of diagnoses recorded in 2000 was estimated as 30 000–41 000.^22^ In women we simulated median (2.5th–97.5th centile) 46 000 (25 000–77 000) symptomatic cases; 48 000–105 000 diagnoses were recorded.^22^ For NHANES, we simulated median (2.5th–97.5th centile) 397 000 (83 000–1149 000) symptomatic cases in men; the number of diagnoses recorded in 2009 was 307 000. We simulated median (2.5th–97.5th centile) 429 000 (259 000–682 000) symptomatic cases in women, and 879 000 diagnoses were recorded.

In the sensitivity analyses we found that relaxing the assumption of equal partnership numbers in men and women led to no meaningful differences in the posterior distributions for transmission probabilities. In a model where partnerships formed only between individuals reporting the same number of partners, we found evidence of higher transmission probabilities in couples reporting fewer partners. Our model using data on partnerships without a condom resulted in posterior distributions shifted to slightly higher transmission probabilities, but the shift was small compared with the width of the distribution.

## Discussion

We have described a new statistical model for inferring the per-partnership transmission probability of a sexually transmitted infection, and have applied it to population-level data on chlamydia from the UK and the USA. Our method provides its estimates with uncertainty, which is crucial for modelling and understanding chlamydia epidemiology and control. Estimates of average per-partnership (as opposed to per-sex-act) transmission probability are valuable for building predictive models of control measures, because data availability means that behavioural models can be parameterized more reliably in terms of number of partnerships than number of sex acts. Our estimates incorporate data from several sources including population-based surveys and make use of information that is often disregarded, contained in the correlation between the number of partners reported and the probability of chlamydia infection.

In the UK we found a male-to-female transmission probability of 32.1% per partnership (95%CrI 18.4–55.9%), which was consistent with the corresponding US result of 34.9% (95%CrI 22.6–54.9%). The posterior for female-to-male transmission probability inferred from the UK data was much more uncertain, with posterior median 21.4% (95%CrI 5.1–67.0%). The equivalent for the US data was lower, but with a narrower and overlapping credible interval: 4.6% (95%CrI 1.0–13.1%).

Posterior predictive checks agreed well with the original data, indicating a well-specified model. The main exception is the partnership number data in women: in both Natsal-2 and NHANES, higher partner numbers are under-reported compared to simulations. Under-reporting of partner numbers by women is a recognized phenomenon which has been widely discussed.^23^ The partnership number distributions may explain the low female-to-male transmission estimated using NHANES. If NHANES respondents reported new partner(s) in the last year, and more than one partner in total, then we took the number of new partners to be one less than the total number of partners: in fact, this proxy is an upper bound, as more than one could have been an existing partner. If the number of partners and hence the contact rate is over-estimated by this proxy then there will be a corresponding reduction in the per-partnership transmission probability.

Katz estimated a male-to-female transmission probability of 39.5% (95%CI 19.3–59.7%) per partnership:^9^ consistent with our estimate. Katz’s estimate for female-to-male transmission probability is 32.3% (95%CI 10.0–54.6%): well within our credible interval for UK data, but barely overlapping for the US estimate. Althaus *et al*.’s ODE-based pair model produced a higher estimated transmission probability per partnership (55.5%, IQR 49.2–62.5%), assuming two partners every 6 months (four per year).^10^ However, they note that their model does not account for heterogeneity in transmissibility of chlamydia, whereas ours allows for differences by sex. We also account for sex differences in chlamydia clearance rate and heterogeneity in partnership turnover rates, which is an important feature in explaining observed partner number distributions.

Our model assumes a closed system at steady state. This assumption is reasonable as the number of people entering and leaving the sexually-active population each year is small compared to the total population, and any changes in the model parameters are slow compared to the dynamics of the system. We have ignored the role of same-sex contacts, but their effect on our estimates is also likely to be small because only people with at least one opposite-sex partner were included in the data. We chose to include people reporting partners of both sexes in our analysis to maximise the amount of data used, and because excluding them ignores their involvement in the heterosexual network and could bias our results.

Another assumption of the analysis is that individuals choose partnerships at random from all the partnerships offered by the opposite sex. Although we know that sexual mixing is to some extent assortative, sensitivity analysis indicates that assortativity would not lead to greatly differing force of infection per contact in people reporting different numbers of partners (see Supplementary Information, available as Supplementary data at *IJE* online). There was some evidence from this analysis of a higher transmission probability in people reporting no new partners, particularly in the NHANES dataset. This could reflect lower condom use or longer partnerships and would be an interesting avenue for further research. However, even if there are qualitative differences between partnerships, leading to heterogeneity in transmission probabilities, this does not invalidate the concept of a single average across all partnerships, which is still a hugely useful quantity for modelling. In a further sensitivity analysis we modelled number of partnerships without a condom, estimated using data from Natsal-2. The posterior distributions suggested that qualitative differences such as condom use may reduce population-average transmission probabilities, but to an extent that is small compared with the uncertainty in the estimates. It might be valuable for sexual behaviour surveys to collect explicit information on the annual number of new partnerships without a condom for parameter inference and predictive modelling, and our sensitivity analysis suggests that our model could be used to infer transmission probabilities from such data.

The evidence synthesis approach that we used can readily incorporate further data as it becomes available, so that improved data collection would allow our analysis to be augmented to improve our estimates. For example, there is particular uncertainty in the proportion of infections that become symptomatic in each sex, and in the clearance rate of untreated infections in men; the latter limiting the precision of the female-to-male transmission probability. We have argued elsewhere that surveillance and screening programmes could be used to collect data on long-term chlamydia clearance in men to inform a more precise estimate of clearance rate.^15^ Additionally, it has been suggested that previous exposure to chlamydia may confer partial immunity,^24^ which would reduce the transmission probability to older and/or more sexually active individuals, who would be more likely to have had a prior infection. Although further empirical study of chlamydia immunology is required, it is interesting that the posterior predictive checks showed that our model tends to under-predict prevalence in those reporting few partners and over-predict in those reporting several partners (Supplementary Figure 4, available as Supplementary data at *IJE* online), which would be consistent with partial immunity in high-risk individuals who are more likely to have been infected before.

In conclusion, it is important to use rigorous parameter estimates in computational models, and to quantify their uncertainty and its effect on conclusions and recommendations. Our method provides such estimates for the probability of chlamydia transmission, and with appropriate data the methods described here could also be applied to other sexually transmitted infections that can be represented using the SIS model. The estimates can be used in transmission modelling to understand the effect of control policies on patterns of prevalence.

## Ethics

This was a secondary analysis of publicly-available data, and no additional ethical approval was required or sought.

## Supplementary data

Supplementary data are available at *IJE* online.

## Funding

J.L. and P.J.W. were supported by the National Institute for Health Research (NIHR) Health Protection Research Unit (HPRU) in Modelling Methodology at Imperial College London in partnership with Public Health England (PHE) (grant number HPRU-2012-10080). P.J.W. was also supported by the NIHR HPRU in Modelling and Health Economics, a partnership between PHE, Imperial College London and LSHTM, for funding (grant number NIHR200908). Additionally, P.J.W. was supported by the MRC Centre for Global Infectious Disease Analysis (grant number MR/R015600/1); this award is jointly funded by the UK Medical Research Council (MRC) and the UK Foreign, Commonwealth & Development Office (FCDO) under the MRC/FCDO Concordat agreement and is also part of the EDCTP2 programme supported by the European Union (EU). M.J.P. was supported by the NIHR Birmingham Biomedical Research Centre at the University Hospitals Birmingham NHS Foundation Trust and the University of Birmingham. This paper presents independent research, and the funders had no role in study design, data collection and analysis, decision to publish, or preparation of the manuscript. The views expressed are those of the authors and not necessarily those of the Department of Health and Social Care, EU, FCDO, MRC, National Health Service, NIHR, or PHE.

## Data Availability

The raw data analysed in this study has been made publicly available by the researchers in question, and can be accessed as described in the References.

## Conflict of Interest

None declared.

## Supplementary Material

dyaa202_Supplementary_Data
