# Supplementary material for: Per-partnership transmission probabilities for Chlamydia trachomatis infection: evidence synthesis of population-based survey data
Source: Int J Epidemiol. 2020 Dec 8;50(2):510–7. doi: 10.1093/ije/dyaa202 (PMC8128448; doi:10.1093/ije/dyaa202)
Supplement: dyaa202_Supplementary_Data [file dyaa202_supplementary_data.pdf]

# Per-partnership transmission probabilities for *Chlamydia trachomatis* infection: Evidence synthesis of population-based survey data – Supplementary Information

Joanna Lewis, Peter J. White and Malcolm J. Price

## Contents

1. Methods ..... 2
   a. Mathematical model ..... 2
   b. Data ..... 3
      i. Clearance of untreated chlamydia infection ..... 3
      ii. Partnership numbers ..... 3
   c. Statistical model ..... 7
      i. Partnership dynamics ..... 7
      ii. Prevalence ..... 8
      iii. Infection clearance rate ..... 8
      iv. Full likelihood ..... 9
   d. Inference and Estimation ..... 10
      i. Priors ..... 10
      ii. Bayesian methods and sampling of posterior distribution ..... 10
      iii. Posterior predictive checks ..... 10
2. Results ..... 11
   a. Posterior parameter distributions ..... 11
   b. Posterior predictive checks ..... 12
      i. Partner number distributions ..... 12
      ii. Infection status ..... 13
      iii. Symptomatic infections ..... 16
   c. Sensitivity Analysis ..... 16
      i. Balancing partnership numbers ..... 16
      ii. Condom use ..... 18
      iii. Assortative mixing ..... 19
3. References ..... 21

## 1. Methods

The aim of the study is to provide a mathematical and statistical model that can be used to infer per-partnership transmission probability from survey data.

### a. Mathematical model

Let each individual *j* experience a force of infection $F_j$, which depends on his or her rate of forming infectious contacts (partnerships). Assume that all women recover from infection at the same rate, $\lambda_f$, and all men recover at the same rate, $\lambda_m$. We use a susceptible-infected-susceptible (SIS) model of infection and recovery (Figure 1). The probability that individual *j* is infected at a given moment is $\pi_j$, and the probability that he or she is susceptible is $1 - \pi_j$.

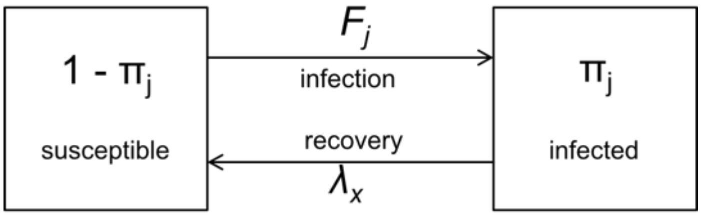


**Figure S1:** Susceptible-infected-susceptible (SIS) model of chlamydia infection and recovery.

Assuming only heterosexual transmission, the force of infection is the rate at which an individual makes contacts with infected members of the opposite sex, multiplied by the per-contact transmission probability. We denote the sex of individual *j* with the symbol *x*, and the opposite sex with the symbol *x′*. The rate of contacting infected members of the opposite sex is $\chi_{xj}$, and the per-contact transmission probability from the opposite sex is $\rho_{x'\to x}$. Then:

$$F_j = \chi_{xj}\rho_{x'\to x}.$$

At steady state, the number of new infections per unit time equals the number of recoveries, so we know also that:

$$F_j(1-\pi_j) = \chi_{xj}\rho_{x'\to x}(1-\pi_j) = \lambda_x\pi_j$$

Hence,

$$\rho_{x'\to x} = \frac{\pi_j}{1-\pi_j}\frac{\lambda_x}{\chi_{xj}}$$

and

$$\frac{\pi_j}{1-\pi_j} = \frac{\chi_{xj}\rho_{x'\to x}}{\lambda_x}$$

The following assumptions are implicit in this argument and are discussed in the main text:

1. Closed system: the number of people entering and leaving the system is negligible.
2. Steady state: prevalence is stable, and force of infection and recovery rate do not change.
3. Identical partnerships: all partnerships have the same risk of transmission, regardless of partnership length and frequency of sex acts.

Our model considers asymptomatic infections; symptomatic infections prompt treatment-seeking and are therefore short-lived and unlikely to cause onward infection or to be detected in population-based surveys.

## b. Data

We infer parameter values in the model by synthesizing data from several sources.

### *i. Clearance of untreated chlamydia infection*

Data informing the clearance rate of untreated chlamydia infection in men and women came from studies in the literature synthesized in previous analyses.[1,2] In each study people found to be infected with chlamydia were re-tested at a later date, having remained untreated in the interim. The number who cleared their infection provides information on the clearance rate. Nine studies in women and eight in men were included, involving a total of 569 women and 165 men. Further details are provided in the original papers describing this analysis.[1,2]

### *ii. Partnership numbers*

We used data on sexual behaviour and chlamydia infection from two population-based studies: the second National Study of Sexual Attitudes and Lifestyles (Natsal-2),[3] and the three National Health and Nutrition Examination Surveys (NHANES) conducted biennially between 2009 and 2014[4]. The ideal data to inform the sexual contact rate would be the number of new sexual partnerships formed in the last year.

In Natsal-2, participants reported their number of opposite-sex partners in the last year and were then asked:

- *Was this [woman/man] a new partner who you had sex with for the first time during the last year?* (if they had reported one partner) or
- *How many of these [women/men] were new partners who you had sex with for the first time during the last year?* (if they had reported more than one partner).

This information was used to inform the distribution of number of new partners in the last year in the Natsal-2 population.

We combined data from the three NHANES conducted between 2009 and 2014 to achieve a larger sample size than would be possible using just one study.[4] Participants were asked:

- *In the past 12 months, with how many [women/men] have you had vaginal sex?* and
- *In the past 12 months, did you have any kind of sex with a person that you never had sex with before?*

We used these two questions to provide a proxy for the number of new partners in the last year according to the following algorithm:

- If a participant stated they had had no new partners in the last year, we took the number of new partners to be zero.
- If a participant stated they had had new partner(s) in the last year, and reported one partner in total, we took the number of new partners to be one.
- If a participant stated they had had new partner(s) in the last year, and reported more than one partner in total, we took the number of new partners to be one less than the total number of partners.

This approach is similar to the use elsewhere of “shifted negative binomial” distributions for modelling partner numbers.[5]

### *iii. Infection status*

The publicly-available data from both Natsal-2 and NHANES also includes chlamydia infection status, diagnosed using nucleic acid amplification tests (NAATs) on urine samples, which provides information on the prevalence of infection in individuals reporting different numbers of partners. Natsal-2 participants were eligible for a urine sample if they were aged 18-44 years and had ever had sex, and a randomly-

selected half of these eligible participants were invited to provide samples. All NHANES participants aged 14-39 years were invited to provide a sample for chlamydia testing, but the publicly-available data excludes 14-17-year-olds.

The raw data on numbers of partnerships reported by susceptible and infected men and women in Natsal-2 and NHANES are provided in Supplementary Tables S1 and S2.

**Table S1:** Raw data from the Second National Study of Sexual Attitudes and Lifestyles (Natsal-2) used to inform the model.

| Number of new partners | Number of Men | | | | | | Chlamydia prevalence in men (95%CI) (%) | Number of Women | | | | | | Chlamydia prevalence in women (95%CI)(%) |
|---|---|---|---|---|---|---|---|---|---|---|---|---|---|---|
| | Unweighted | | | Weighted | | | | Unweighted | | | Weighted | | | |
| | Uninfected | Infected | Total | Uninfected | Infected | Total | | Uninfected | Infected | Total | Uninfected | Infected | Total | |
| 0 | 784 | 9 | 793 | 1063.78 | 13.57 | 1077.35 | 1.3 (0.5, 2.5) | 1346 | 17 | 1363 | 1210.19 | 11.38 | 1221.57 | 0.9 (0.5, 1.5) |
| 1 | 243 | 6 | 249 | 266.15 | 6.57 | 272.72 | 2.4 (0.8, 5.6) | 309 | 9 | 318 | 213.90 | 5.11 | 219.01 | 2.3 (1.0, 4.7) |
| 2 | 98 | 4 | 102 | 99.39 | 4.91 | 104.30 | 4.7 (1.1, 12.5) | 76 | 5 | 81 | 55.64 | 2.85 | 58.49 | 4.9 ( 1.4, 11.6) |
| 3 | 51 | 3 | 54 | 48.32 | 4.62 | 52.94 | 8.7 (1.5, 25.1) | 27 | 2 | 29 | 16.57 | 1.82 | 18.39 | 9.9 (1.0, 33.2) |
| 4 | 18 | 2 | 20 | 16.79 | 3.05 | 19.84 | 15.4 (1.3, 50.0) | 18 | 0 | 18 | 13.40 | 0 | 13.40 | 0* |
| 5 | 14 | 1 | 15 | 13.55 | 0.94 | 14.50 | 6.5 (0.1, 32.8) | 9 | 2 | 11 | 7.33 | 0.86 | 8.19 | 10.5 (0.9, 36.2) |
| 6 | 8 | 1 | 9 | 8.88 | 1.94 | 10.83 | 18.0 (0.4, 67.7) | 3 | 0 | 3 | 2.04 | 0 | 2.04 | - |
| 7 | 6 | 0 | 6 | 7.92 | 0 | 7.92 | 0* | 2 | 0 | 2 | 1.06 | 0 | 1.06 | - |
| 8 | 1 | 0 | 1 | 1.62 | 0 | 1.62 | 0* | 1 | 0 | 1 | 0.48 | 0 | 0.48 | - |
| 9 | 5 | 2 | 7 | 5.68 | 1.51 | 7.19 | 21.0 (2.1, 60.6) | 1 | 0 | 1 | 1.26 | 0 | 1.26 | - |
| 10 | 3 | 1 | 4 | 4.09 | 0.58 | 4.67 | 12.4 (0.0, 1.0) | 1 | 1 | 2 | 0.48 | 0.25 | 0.73 | 34.1‡ |
| 11 | 1 | 0 | 1 | 1.76 | 0 | 1.76 | 0* | 0 | 1 | 1 | 0 | 0.62 | 0.62 | 100* |
| 12 | 3 | 0 | 3 | 4.46 | 0 | 4.46 | 0* | 0 | 0 | 0 | 0 | 0 | 0 | - |
| 13 | 1 | 0 | 1 | 0.99 | 0 | 0.99 | 0* | 1 | 0 | 1 | 0.81 | 0 | 0.81 | 0* |
| 15 | 1 | 0 | 1 | 1.16 | 0 | 1.16 | 0* | 0 | 0 | 0 | 0 | 0 | 0 | - |
| 19 | 1 | 0 | 1 | 0.78 | 0 | 0.78 | 0* | 0 | 0 | 0 | 0 | 0 | 0 | - |
| 20 | 0 | 0 | 0 | 0 | 0 | 0 | - | 2 | 0 | 2 | 1.91 | 0 | 1.91 | 0* |
| 36 | 0 | 0 | 0 | 0 | 0 | 0 | - | 1 | 0 | 1 | 0.46 | 0 | 0.46 | 0* |
| 39 | 0 | 0 | 0 | 0 | 0 | 0 | - | 1 | 0 | 1 | 0.44 | 0 | 0.44 | 0* |
| 55 | 1 | 0 | 1 | 0.44 | 0 | 0.44 | 0* | 0 | 0 | 0 | 0 | 0 | 0 | - |
| **Overall mean number of partners** | 0.85 | 2.41 | 0.89 | 0.71 | 2.07 | 0.74 | **Overall prevalence in men:** 2.38 (1.52, 3.55) | 1.03 | 1.51 | 1.04 | 0.95 | 1.31 | 0.96 | **Overall prevalence in women:** 1.48 (1.01, 2.08) |

CI: confidence interval

*Confidence intervals cannot be calculated where 0% or 100% of individuals were infected.

‡A confidence interval could not be calculated because of the small number of individuals.

**Table S2:** Raw data from the National Health and Nutrition Examination Surveys (NHANES; 2009-2014 combined) used to inform the model.

| Number of new partners | Number of Men | | | | | | Chlamydia prevalence in men (95%CI) (%) | Number of Women | | | | | | Chlamydia prevalence in women (95%CI)(%) |
|---|---|---|---|---|---|---|---|---|---|---|---|---|---|---|
| | Unweighted | | | Weighted | | | | Unweighted | | | Weighted | | | |
| | Uninfected | Infected | Total | Uninfected | Infected | Total | | Uninfected | Infected | Total | Uninfected | Infected | Total | |
| 0 | 1617 | 29 | 1646 | 1662.23 | 25.61 | 1687.84 | 1.5 (0.9, 2.3) | 1901 | 43 | 1944 | 1898.59 | 34.05 | 1932.65 | 1.8 (1.2, 2.5) |
| 1 | 306 | 12 | 318 | 330.54 | 10.92 | 341.46 | 3.2 (1.4, 6.3) | 291 | 18 | 309 | 289.92 | 10.79 | 300.71 | 3.6 (2.0, 5.9) |
| 2 | 123 | 4 | 127 | 109.99 | 2.99 | 112.98 | 2.6 (0.6, 7.1) | 78 | 7 | 85 | 78.20 | 5.21 | 83.42 | 6.3 (1.8, 14.9) |
| 3 | 72 | 2 | 74 | 61.48 | 0.83 | 62.31 | 1.3 (0.1, 5.5) | 34 | 2 | 36 | 31.85 | 1.20 | 33.05 | 3.6 (0.1, 18.5) |
| 4 | 58 | 2 | 60 | 55.29 | 0.65 | 55.94 | 1.2 (0.1, 4.9) | 17 | 0 | 17 | 15.40 | 0 | 15.40 | 0* |
| 5 | 19 | 2 | 21 | 23.46 | 0.72 | 24.18 | 3.0 (0.0, 92.3) | 15 | 1 | 16 | 14.50 | 0.44 | 14.94 | 2.9 (0.0, 93.5) |
| 6 | 23 | 1 | 24 | 22.14 | 0.55 | 22.69 | 2.4 (0.0, 19.3) | 4 | 1 | 5 | 4.78 | 0.44 | 5.22 | 8.43[‡] |
| 7 | 13 | 0 | 13 | 14.68 | 0 | 14.68 | 0* | 8 | 0 | 8 | 8.31 | 0 | 8.31 | 0* |
| 8 | 11 | 0 | 11 | 9.24 | 0 | 9.24 | 0* | 3 | 1 | 4 | 5.22 | 1.35 | 6.57 | 20.6[‡] |
| 9 | 19 | 1 | 20 | 17.35 | 0.62 | 17.97 | 3.5 (0.0, 98.8) | 3 | 0 | 3 | 1.91 | 0 | 1.91 | 0* |
| 10 | 3 | 0 | 3 | 2.89 | 0 | 2.89 | 0* | 2 | 0 | 2 | 0.98 | 0 | 0.98 | 0* |
| 11 | 8 | 0 | 8 | 6.41 | 0 | 6.41 | 0* | 7 | 0 | 7 | 4.25 | 0 | 4.25 | 0* |
| 12 | 5 | 0 | 5 | 4.31 | 0 | 4.31 | 0* | 0 | 1 | 1 | 0 | 0.62 | 0.62 | 0* |
| 13 | 3 | 0 | 3 | 3.21 | 0 | 3.21 | 0* | 0 | 0 | 0 | 0 | 0 | 0 | - |
| 14 | 8 | 1 | 9 | 5.09 | 0.53 | 5.62 | 9.4[‡] | 1 | 0 | 1 | 0.42 | 0 | 0.42 | 0* |
| 17 | 3 | 1 | 4 | 2.72 | 0.44 | 3.16 | 13.9[‡] | 1 | 0 | 1 | 0.69 | 0 | 0.69 | 0* |
| 18 | 3 | 0 | 3 | 2.36 | 0 | 2.36 | 0* | 0 | 0 | 0 | 0 | 0 | 0 | - |
| 19 | 6 | 0 | 6 | 3.99 | 0 | 3.99 | 0* | 0 | 1 | 1 | 0 | 0.39 | 0.39 | 100* |
| 21 | 2 | 0 | 2 | 2.90 | 0 | 2.90 | 0* | 0 | 0 | 0 | 0 | 0 | 0 | - |
| 23 | 0 | 0 | 0 | 0 | 0 | 0 | - | 1 | 0 | 1 | 0.82 | 0 | 0.82 | 0* |
| 24 | 3 | 1 | 4 | 3.36 | 0.52 | 3.89 | 13.5[‡] | 2 | 0 | 2 | 4.13 | 0 | 4.13 | 0* |
| 29 | 2 | 0 | 2 | 1.60 | 0 | 1.60 | 0* | 1 | 0 | 1 | 0.60 | 0 | 0.60 | 0* |
| 34 | 1 | 0 | 1 | 0.80 | 0 | 0.80 | 0* | 1 | 0 | 1 | 1.24 | 0 | 1.24 | 0* |
| 38 | 1 | 0 | 1 | 0.62 | 0 | 0.62 | 0* | 0 | 0 | 0 | 0 | 0 | 0 | - |
| 39 | 1 | 0 | 1 | 0.36 | 0 | 0.36 | 0* | 0 | 0 | 0 | 0 | 0 | 0 | - |
| 44 | 1 | 0 | 1 | 2.11 | 0 | 2.11 | 0* | 0 | 0 | 0 | 0 | 0 | 0 | - |
| 49 | 4 | 0 | 4 | 5.52 | 0 | 5.52 | 0* | 1 | 0 | 1 | 1.60 | 0 | 1.60 | 0* |
| 78 | 1 | 0 | 1 | 0.90 | 0 | 0.90 | 0* | 0 | 0 | 0 | 0 | 0 | 0 | - |
| 79 | 1 | 0 | 1 | 0.64 | 0 | 0.64 | 0* | 0 | 0 | 0 | 0 | 0 | 0 | - |
| 89 | 0 | 0 | 0 | 0 | 0 | 0 | - | 1 | 0 | 1 | 2.22 | 0 | 2.22 | 0* |
| 99 | 1 | 1 | 2 | 0.75 | 0.52 | 1.27 | 41.2[‡] | 0 | 0 | 0 | 0 | 0 | 0 | - |
| **Overall mean number of partners** | 1.26 | 3.74 | 1.31 | 1.18 | 2.53 | 1.20 | **Overall prevalence in men:** 1.87 (1.33, 2.56) | 0.52 | 1.17 | 0.54 | 0.57 | 1.01 | 0.58 | **Overall prevalence in women:** 2.25 (1.65, 3.00) |

CI: confidence interval

*Confidence intervals cannot be calculated where 0% or 100% of individuals were infected.

[‡]A confidence interval could not be calculated because of the small number of individuals.

### c. Statistical model

We conducted a Bayesian evidence synthesis using data from the sources described to construct a likelihood. This was combined with appropriate priors to provide a posterior distribution for the model parameters.

#### *i. Partnership dynamics*

We used negative binomial distributions to model the estimated numbers of new partners reported in the last year by men and women. A negative binomial distribution with size $\alpha$ and mean $\mu$ can arise as a mixture of Poisson distributions, where the mixing distribution for the Poisson rate is a Gamma distribution with shape $\alpha$ and rate $\frac{\mu}{\alpha}$. Formally, let the number of new partners reported by individual *j* be represented by the random variable $N_j$ which has a Poisson distribution with rate $\sigma_j$:

$$N_j \sim Poisson(\sigma_j),$$

so that

$$P(n_j|\sigma_j) = P_{Poisson}(n_j|\sigma_j) = \frac{e^{-\sigma_j}{\sigma_j}^{n_j}}{n_j!}$$

Now, let the partner change rate be a random variable having a Gamma distribution with shape $\alpha_j$ and rate $\beta_j = \frac{\mu_j}{\alpha_j}$:

$$\sigma_j \sim Gamma(\alpha_j, \beta_j)$$

so that

$$P(\sigma_j|\alpha_j, \beta_j) = P_{Gamma}(\sigma_j|\alpha_j, \beta_j) = \frac{(\beta_j)^{\alpha_j}}{\Gamma(\alpha_j)} \sigma_j^{(\alpha_j - 1)} e^{-\sigma_j \beta_j}.$$

It can be shown[6] by integrating over the Poisson rate $\sigma_j$ that $N_j$ has a negative binomial distribution with size $\alpha_j$ and mean $\mu_j = {\alpha_j}/{\beta_j}$:

$$N_j \sim NB(\mu_j, \alpha_j)$$

$$P(n_j|\mu_j, \alpha_j) = P_{NB}(n_j|\mu_j, \alpha_j) = \binom{n_j + \alpha_j - 1}{n_j} \left(\frac{\mu_j}{\mu_j + \alpha_j}\right)^{n_j} \left(\frac{\alpha_j}{\mu_j + \alpha_j}\right)^{\alpha_j}$$

In our model, the shape and rate depend on the sex of the individual:

$$(\alpha_j, \beta_j) = \begin{cases} (\alpha_m, \beta_m) & \text{for men} \\ (\alpha_f, \beta_f) & \text{for women} \end{cases}$$

As we are considering heterosexual transmission, the expected number of partnerships per man must equal the expected number of partnerships per woman, so we constrain the negative binomial partnership number distributions in men and women to have the same mean:

$${\alpha_m}/{\beta_m} = {\alpha_f}/{\beta_f} = \mu.$$

The Gamma distribution is the conjugate prior for the Poisson. Given that we observe $n_j$ new partnerships in a year in individual *j*, we can "update" our knowledge of the partner change rate in individual *j* and say that

$$\sigma_j \sim Gamma(\alpha_j + n_j, \beta_j + 1)$$

(See [6] for a full discussion of conjugate priors, including the Poisson model.)

### *ii. Prevalence*

As described above, the probability that individual *j* is infected with chlamydia is a function of the Poisson rate of forming partnerships with infected people ($\chi_{xj}$), the per-partnership transmission probability ($\rho_{x'\to x}$), and the clearance rate ($\lambda_x$):

$$\frac{\pi_j}{1-\pi_j} = \frac{\chi_{xj}\rho_{x'\to x}}{\lambda_x} \qquad (1)$$

The rate of individual *j* forming infectious contacts, $\chi_{xj}$, equals the rate of forming contacts, $\sigma_j$, multiplied by the proportion of contacts offered by the opposite sex that are infectious, $\pi_p^{x'}$:

$$\chi_{xj} = \sigma_j \pi_p^{x'} \qquad (2)$$

$\pi_p^{x'}$ is calculated by integrating (numerically) the product of prevalence and expected number of partnerships formed, over all possible partner change rates in sex *x'*, and then dividing by the total expected number of partnerships formed, $\mu_{x'} = \mu$:

$$\pi_p^{x'} = \frac{1}{\mu}\int_{\sigma=0}^{\infty} P(\sigma|\mu,\alpha_{x'})\frac{\sigma\pi_p^{x}\rho_{x\to x'}}{\sigma\pi_p^{x}\rho_{x\to x'} + \lambda_x}\sigma d\sigma$$

Substituting (2) into (1), the probability that an individual *j* is infected, $\pi_j$, therefore fulfills the equality:

$$\frac{\pi_j}{1-\pi_j} = \frac{\sigma_j\pi_p^{x'}\rho_{x'\to x}}{\lambda_x}$$

$$\pi_j = \frac{1}{1+z_j}$$

where

$$z_j = \frac{\lambda_x}{\sigma_j\pi_p^{x'}\rho_{x'\to x}}$$

For individual *j*, the exact value of $\sigma_j$ is not known, but the reported number of new partners, $n_j$, provides some information, allowing us to update our Gamma prior as described above. The expected prevalence in individuals reporting $n_j$ partners is calculated by integrating the product of prevalence and the updated Gamma probability density for individual *j*:

$$\begin{aligned}\pi_j &= \int_{\sigma=0}^{\infty} P_{Gamma}(\sigma|\alpha_j+n_j,\beta_j+1)\frac{1}{1+z(\sigma_j)}d\sigma \\ &= \frac{(\beta_j+1)^{\alpha_j+n_j}}{\Gamma(\alpha_j+n_j)}\int_{\sigma=0}^{\infty}\frac{\sigma^{\alpha_j+n_j}e^{-(\beta_j+1)\sigma}}{\sigma+\lambda_x/\pi_p^{x'}\rho_{x'\to x}}d\sigma\end{aligned}$$

The infection status of *j* has a Bernoulli distribution with parameter $\pi_j$:

$$P(\delta_j|\pi_j) = P_{Bernoulli}(\delta_j|\pi_j) = \begin{cases}\pi_j & \delta_j = 1\\ 1-\pi_j & \delta_j = 0\end{cases}$$

where

$$\delta_j = \begin{cases}1 & \text{if } j \text{ is infected}\\ 0 & \text{if } j \text{ is uninfected}\end{cases}$$

### *iii. Infection clearance rate*

We modelled immunological clearance of infection using the parameter $\lambda_x$. The statistical model is described elsewhere,[1] and allows for two courses of infection: fast- or slow-clearing. A proportion *p* of

incident infections clear fast, and the remainder, 1 – $p$, clear slow. In this analysis we assume that only the slow-clearing infections last long enough to be detected in population-based studies. The clearance rate (denoted $\lambda_x$ below) is therefore equal to the slow clearance rate in the clearance model, and the transmission probability we estimate is the probability that an infection is transmitted and then follows the slow-clearing course. The parameter values are inferred from published observational data in men and women[1,2].

In the absence of data on the rates of testing and treating for asymptomatic chlamydia infection at the time of Natsal-2 and NHANES, we were not able to account in our model for chlamydia clearance via treatment of asymptomatic infections. We investigated the results of this decision in our predictive checks (see below).

### *iv. Full likelihood*

The full set of model parameters is $\{\mu, \beta, \sigma, p, \lambda, \psi, A\}$, where $A = {}^{F_j}/_{\sigma_j} = \pi_p^{x'}\rho_{x'\to x}$ for transmission from $x'$ to $x$ or $\pi_p^x \rho_{x\to x'}$ for transmission from $x$ to $x'$. From these we derive the parameters $\{\alpha, \pi, \pi_p, \rho\}$. The meaning of each symbol is summarized in Table S3.

**Table S3:** Summary of symbols used to describe the model.

| Symbol | Description |
|---|---|
| $\mu$ | Mean number of new partnerships per person. |
| $\beta = (\beta_m, \beta_f)$ | Rate parameters for gamma distributions |
| $\sigma = (\sigma_1, \sigma_2, \dots)$ | Poisson rates of partnership formation. |
| $p = (p_m, p_f)$ | Proportion of infections in men and women which are fast-clearing. |
| $\lambda = (\lambda_m, \lambda_f)$ | Clearance rate of slow-clearing infections |
| $\psi$ | Sensitivity of culture diagnosis methods (for the clearance rate model). |
| $A = (A_{f\to m}, A_{m\to f})$ | Per-partnership prevalence, multiplied by per-partnership transmission probability. |
| $\alpha = (\alpha_m, \alpha_f)$ | Shape parameters for gamma distributions. |
| $\pi = (\pi_1, \pi_2, \dots)$ | Expected chlamydia prevalence in each individual. |
| $\pi_p = (\pi_p^m, \pi_p^f)$ | Proportion of all partnerships in which the man/woman is infected. |
| $\rho = (\rho_{m\to f}, \rho_{f\to m})$ | Per-partnership transmission probability from an infected man/woman to a susceptible woman/man. |

Survey weights $w_i$ are incorporated by multiplying the relevant component of the log-likelihood by the weight. The log-likelihood of the data is given by:

$$L = L_{turnover} + L_{clearance} + L_{infection}$$

where:

- $L_{turnover}$ is the log-likelihood associated with the partnership turnover data in men and women.
$$L_{turnover} = L_{turnover}^m + L_{turnover}^f = \sum_m w_j \times P_{NB}(n_j|\alpha_m, \beta_m) + \sum_f w_j \times P_{NB}(n_j|\alpha_f, \beta_f)$$

- $L_{clearance}$ is the log-likelihood associated with the clearance data:

$$L_{clearance} = \sum_{data} P_{binomial}(r|n_{test}, \theta)$$

where $n_{test}$ is the number of people tested for each data point, $r$ is the number who had cleared their infection and $\vartheta$ is the proportion expected to clear the infection (full details provided elsewhere[1]).

- $L_{prevalence}$ is the log-likelihood associated with the prevalence data in men and women reporting different numbers of partners:

$$L_{prevalence} = \sum_{m} w_j P_{Bernoulli}(\delta_j|\pi_j) + \sum_{f} w_j P_{Bernoulli}(\delta_j|\pi_j)$$

### d. Inference and Estimation

#### *i. Priors*

Prior distributions for the parameters were as follows:

$\mu \sim$ Exponential$(0.1)$ (uninformative)
$\beta \sim$ Exponential$(0.1)$ (uninformative)
$p \sim$ Beta$(1,1)$ (uninformative)
$\lambda_{slow} \sim$ Exponential$(0.001)$ (uninformative)
$\psi \sim$ Beta$(78,8)$ (based on studies comparing test performance[7])
$A \sim$ Exponential$(0.001)$ (uninformative)

#### *ii. Bayesian methods and sampling of posterior distribution*

Estimation was carried out by sampling from the posterior using a Markov chain Monte Carlo (MCMC) algorithm implemented in the Stan software,[8] within the R environment.[9] The data, Stan model file and R scripts used for handling input and results are all available online at https://github.com/mrc-ide/ct_transmission_prob. MCMC estimation is carried out by drawing thousands of samples from the joint posterior distribution. We ran four chains for 2000 iterations each, discarding the first 1000 "warmup" iterations of each chain. The results reported below are summary means, medians and credible intervals of the marginal distributions from this sampled joint posterior.

#### *iii. Posterior predictive checks*

We carried out graphical posterior predictive checks[6] to check the fit of the model. We simulated values for the data (number of partners and infection status for each individual), using each sample from the joint posterior distribution. The simulated data were compared to observed data to look for any systematic differences.

We expect that a proportion $\phi_x$ of incident chlamydia infections in sex *x* will cause symptoms that prompt testing and treatment, while the remaining $1 - \phi_x$ are asymptomatic. As noted above, our model considers *asymptomatic* infections, so the modelled force of infection represents the force of asymptomatic infection. The force of *symptomatic* infection is $\frac{\phi_x}{1-\phi_x}$ times the force of asymptomatic infection, and we expect to observe symptomatic diagnoses in the population at this per-person rate. We used prior distributions for $\phi$ ($\phi_m \sim Beta(11, 5)$; $\phi_f \sim Beta(27, 90)$ [7]), the posteriors for force of infection, and the population size, to simulate the annual number of symptomatic diagnoses.

## 2. Results

### a. Posterior parameter distributions

**Table S4:** Summary of posterior distributions for model parameters, inferred using data from the second National Study of Sexual Attitudes and Lifestyles (Natsal-2) and National Health and Nutrition Examination Surveys (NHANES). The first six parameters were sampled directly; the last three were calculated from the first six, as described in the text.

| Parameter | Natsal | | | | NHANES | | | |
|---|---|---|---|---|---|---|---|---|
| | Men | | Women | | Men | | Women | |
| | Mean | Median (95% CrI) | Mean | Median (95% CrI) | Mean | Median (95% CrI) | Mean | Median (95% CrI) |
| $\mu$ (Mean partnerships) | 0.593 | 0.592 (0.545, 0.646) | Shared parameter | | 0.922 | 0.921 (0.848, 1.001) | Shared parameter | |
| $\beta$ (Rate parameter for gamma distribution) | 0.512 | 0.510 (0.437, 0.597) | 0.366 | 0.364 (0.291, 0.453) | 0.176 | 0.176 (0.156, 0.198) | 0.137 | 0.136, (0.116, 0.160) |
| $p$ (Proportion of infections fast-clearing) | 0.314 | 0.314 (0.208, 0.423) | 0.206 | 0.206 (0.150, 0.266) | 0.316 | 0.316 (0.209, 0.430) | 0.207 | 0.207 (0.152, 0.267) |
| $\lambda$ (year$^{-1}$) (Slow clearance rate) | 0.642 | 0.571 (0.144, 1.54) | 0.737 | 0.735 (0.601, 0.884) | 0.634 | 0.566 (0.122, 1.512) | 0.735 | 0.733 (0.600, 0.883) |
| $\psi$ (Sensitivity of culture diagnosis*) | 0.911 | 0.912 (0.860, 0.953) | Shared parameter | | 0.911 | 0.912 (0.859, 0.954) | Shared parameter | |
| $A = (A_{f\to m}, A_{m\to f})$ ($\pi_p \times \rho$; see below) | 0.025 | 0.022 (0.005, 0.062) | 0.028 | 0.028 (0.018, 0.043) | 0.014 | 0.012 (0.003, 0.033) | 0.040 | 0.039 (0.028, 0.056) |
| $\alpha$ (Shape parameter for gamma distribution) | 0.303 | 0.303 (0.262, 0.350) | 0.217 | 0.215 (0.179, 0.261) | 0.162 | 0.162 (0.146, 0.179) | 0.126 | 0.126 (0.111, 0.142) |
| $\pi_p$ (Proportion of all partnerships infected.) | 0.087 | 0.086 (0.062, 0.115) | 0.105 | 0.104 (0.069, 0.148) | 0.114 | 0.113 (0.084, 0.147) | 0.261 | 0.261 (0.207, 0.317) |
| $\rho = (\rho_{f\to m}, \rho_{m\to f})$ (Per-partnership transmission probability) | 0.252 | 0.214 (0.051, 0.670) | 0.334 | 0.321 (0.184, 0.559) | 0.052 | 0.046 (0.010, 0.131) | 0.358 | 0.349 (0.226, 0.549) |

CrI: credible interval

*Culture sensitivity at re-testing for chlamydia clearance, in people previously diagnosed by culture.[1]

## b. Posterior predictive checks

### *i. Partner number distributions*

Figure S2 illustrates the model's agreement with partnership number data, showing the actual and simulated proportions of men and women who reported each number of partners. Transparent grey circle markers represent simulations from the posterior distributions; lines show the 50th (solid) and 2.5th/97.5th (dashed) centiles of the simulations, and red crosses show the data. For a perfect model and completely accurate reporting of the data, we would expect the dashed lines to enclose 95% of data points.

In both studies, the partnership numbers simulated in men generally agreed well with the data. The predictive properties were less good in women, with under-reporting of high partner numbers compared to simulations. If the average number of partnerships formed by men and women were allowed to differ then the agreement between simulations and data was improved and the posterior distributions for transmission probability remained similar. In our model we chose to constrain the average number in men and women to be equal because this is a necessary condition in reality.

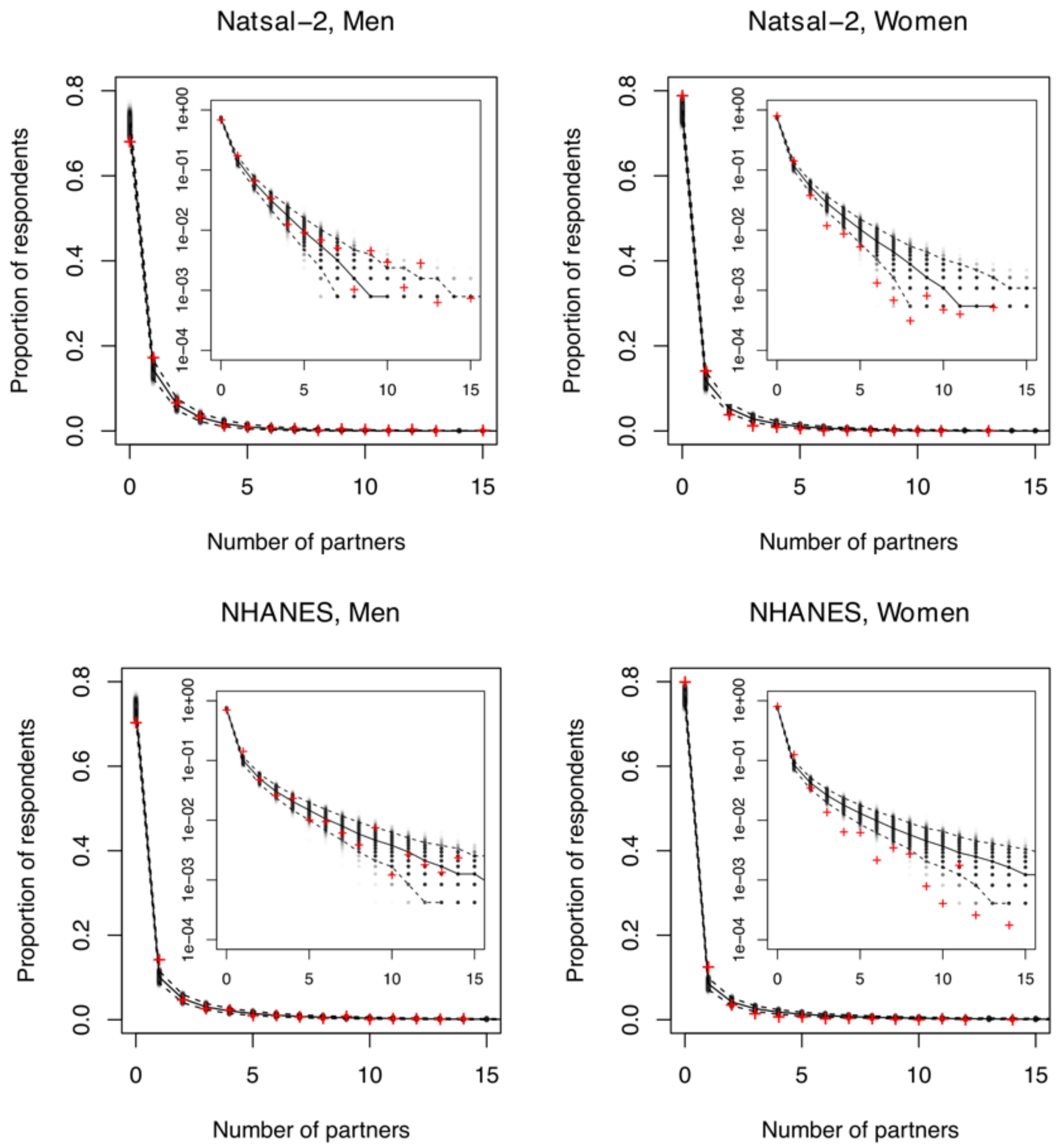


**Figure S2:** Simulated (grey) and observed (red) proportions of men (left) and women (right) reporting different numbers of new partners in the last year in the second National Study of Sexual Attitudes and Lifestyles (Natsal-2; top) and National Health and Nutrition Examination Studies (NHANES) 2009-2014 (bottom). The main graph in each panel uses a linear scale on the y-axis, and the inset shows the same information but on a log scale. Simulations are shown using transparent grey markers, so that several

superimposed markers appear as a darker grey. The solid and dashed lines show the 2.5th, 50th and 97.5th centiles of the simulations. The observed data shown takes into account the survey weights.

### ii. *Infection status*

We checked the predictive properties of the infection model by using each sampled parameter set to simulate infection status in each survey participant, given their reported number of partners. In Figures S3 (Natsal-2) and S4 (NHANES), each transparent grey marker shows simulated prevalence among the participants reporting a given number of partners, which agreed well with the observed data. Only a small number of participants reported the highest numbers of partners (see bar graphs in lower panels), so only a few levels of prevalence were possible in those with several partners. For example, one man in Natsal-2 reported 19 partners, so simulated prevalence could only be 0 (one man, uninfected) or 1 (one man, infected).

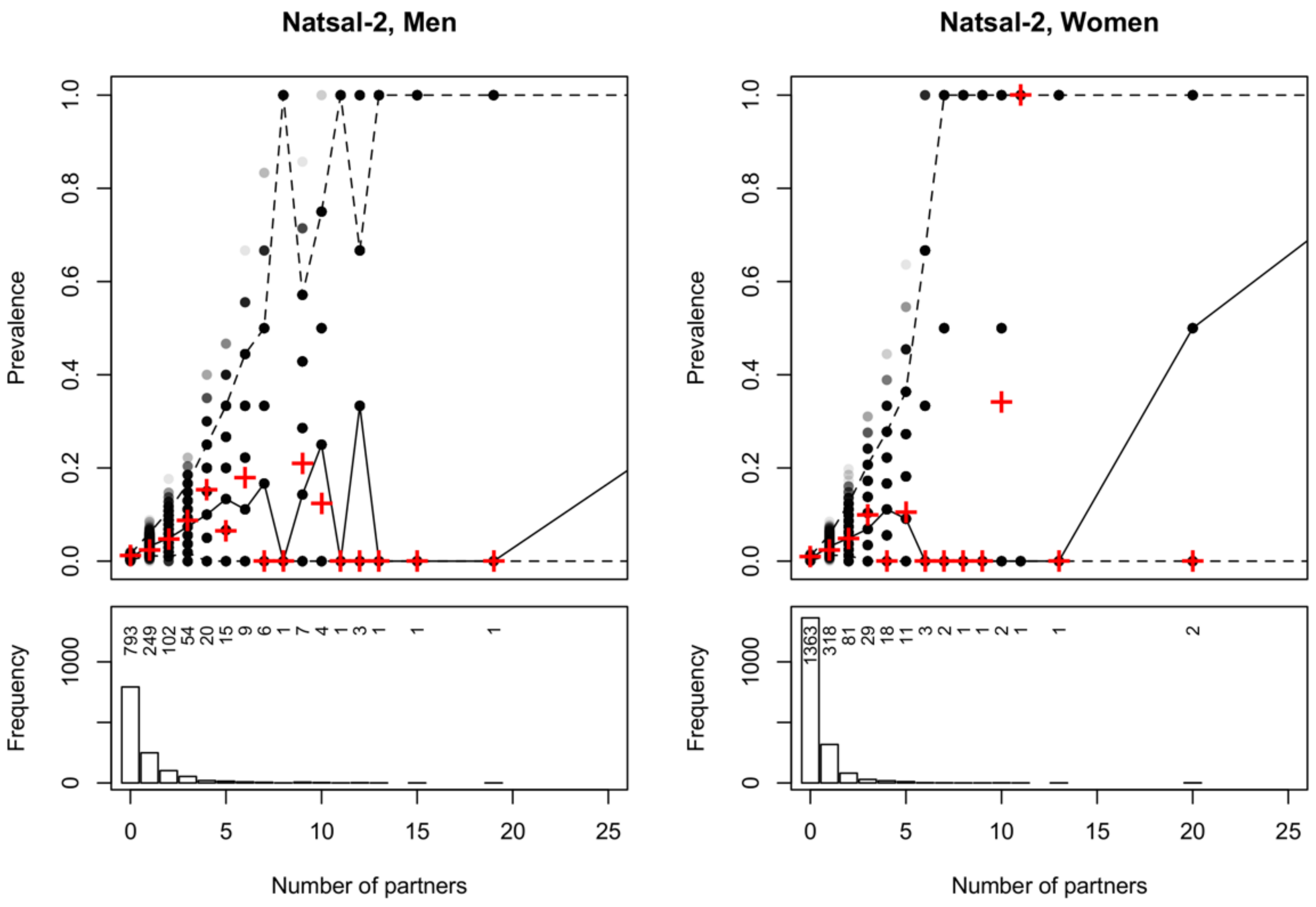


**Figure S3:** Simulated (grey) and observed (red) chlamydia prevalence (y-axis) in men and women reporting different numbers of new partners in the last year (x-axis) in the second National Study of Sexual Attitudes and Lifestyles (Natsal-2). Simulations are shown using transparent grey markers, so that several superimposed markers appear as a darker grey. The solid and dashed lines join the 2.5th, 50th and 97.5th centiles of the simulations. The observed data takes into account the survey weights. Bar charts below each plot show the (unweighted) number of survey participants reporting each number of partnerships.

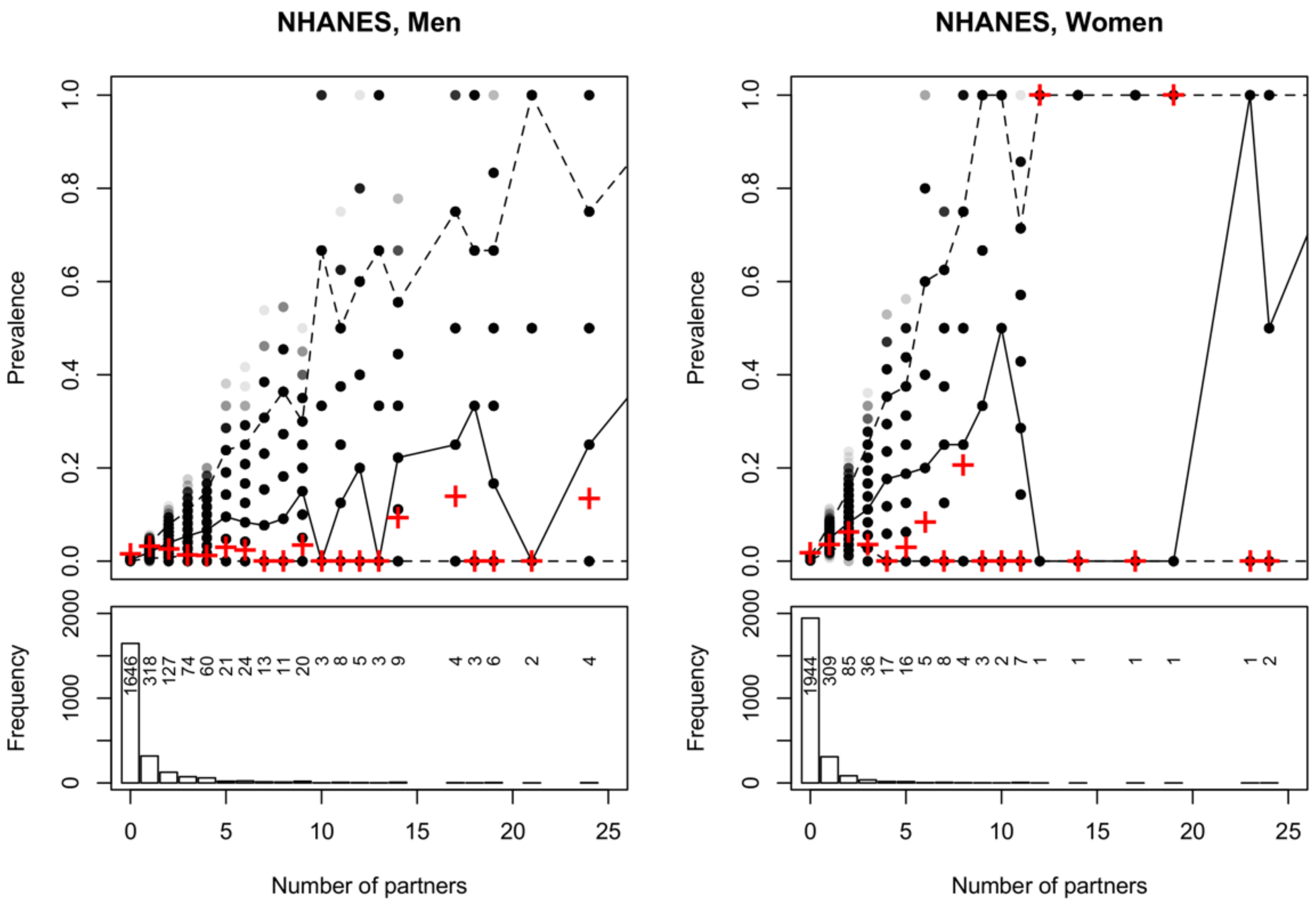


**Figure S4:** Simulated (grey) and observed (red) chlamydia prevalence (y-axis) in men and women reporting different numbers of new partners in the last year (x-axis) in the National Health and Nutrition Examination Studies (NHANES). Simulations are shown using transparent grey markers, so that several superimposed markers appear as a darker grey. The solid and dashed lines join the 2.5th, 50th and 97.5th centiles of the simulations. The observed data takes into account the survey weights. Bar charts below each plot show the (unweighted) number of survey participants reporting each number of partnerships.

### *iii. Symptomatic infections*

Table S5 shows the median and central 95% range of simulated numbers of symptomatic chlamydia cases, based on our posterior distributions and the male and female populations of England aged 15-44 in 2000 (Natsal-2), or the US aged 15-39 in 2009 (NHANES). For comparison, we also report the number of diagnoses recorded in surveillance systems covering approximately the same times and locations. In men in both studies and women in Natsal-2 the range of our simulations overlapped with the range from surveillance, suggesting that most of the observed diagnoses can be accounted for by treatment-seeking in response to symptoms, and that few additional diagnoses were made as a result of asymptomatic testing. In women in NHANES, more diagnoses were observed than we expected to be sought by symptomatic cases alone, so it seems likely that there was additional testing of asymptomatic women which would merit further empirical investigation.

**Table S5:** Numbers of symptomatic chlamydia cases simulated using posterior parameter distributions inferred using Natsal-2 and NHANES data, and diagnoses recorded in surveillance systems covering approximately the same times and locations. For comparison to Natsal-2 we used diagnosis rate ranges in 15-44-year-olds in 2000,[10] and for NHANES we used the range of recorded diagnoses over the years 2009-2014.[11]

| Survey | Group | Simulated symptomatic cases (1000s; median and 95% CrI) | Observed diagnoses (1000s) |
|---|---|---|---|
| Natsal-2 | Men aged 15-44 years | 109 (25-327) | 30-41 |
| | Women aged 15-44 years | 46 (25-77) | 48-105 |
| NHANES | Men aged 15-39 years | 397 (83-1149) | 307-398 |
| | Women aged 15-39 years | 429 (259-682) | 879-981 |

## c. Sensitivity Analysis

### *i. Balancing partnership numbers*

We tested the effect of constraining the mean numbers to be equal by repeating the analysis, relaxing the constraint of equal mean partnership number in men and women (see online code). Figure S5 illustrates this model's agreement with partnership number data. In both studies the agreement between simulations and observations is improved compared to the constrained model, especially in women, but more than 5% of observations still fell outside the 95% prediction interval. Using Natsal-2, the posterior median (95%CrI) for the mean number of new partners per year in men was 0.75 (0.67-0.83) and in women was 0.40 (0.35-0.45). Inferred transmission probabilities were 32.4% (18.4-55.5)% (male-to-female) and 26.2% (5.8-84.8)% (female-to-male). Using NHANES, the inferred mean number of partners in men was 1.10 (1.08-1.33) and in women was 0.58 (0.52-0.66). Transmission probabilities were 31.3% (20.4-48.7)% (male-to-female) and 6.3% (1.4-18.0)% (female-to-male). Therefore, constraining the mean number of partnerships to be equal did not materially change the posterior distributions for transmission probabilities.

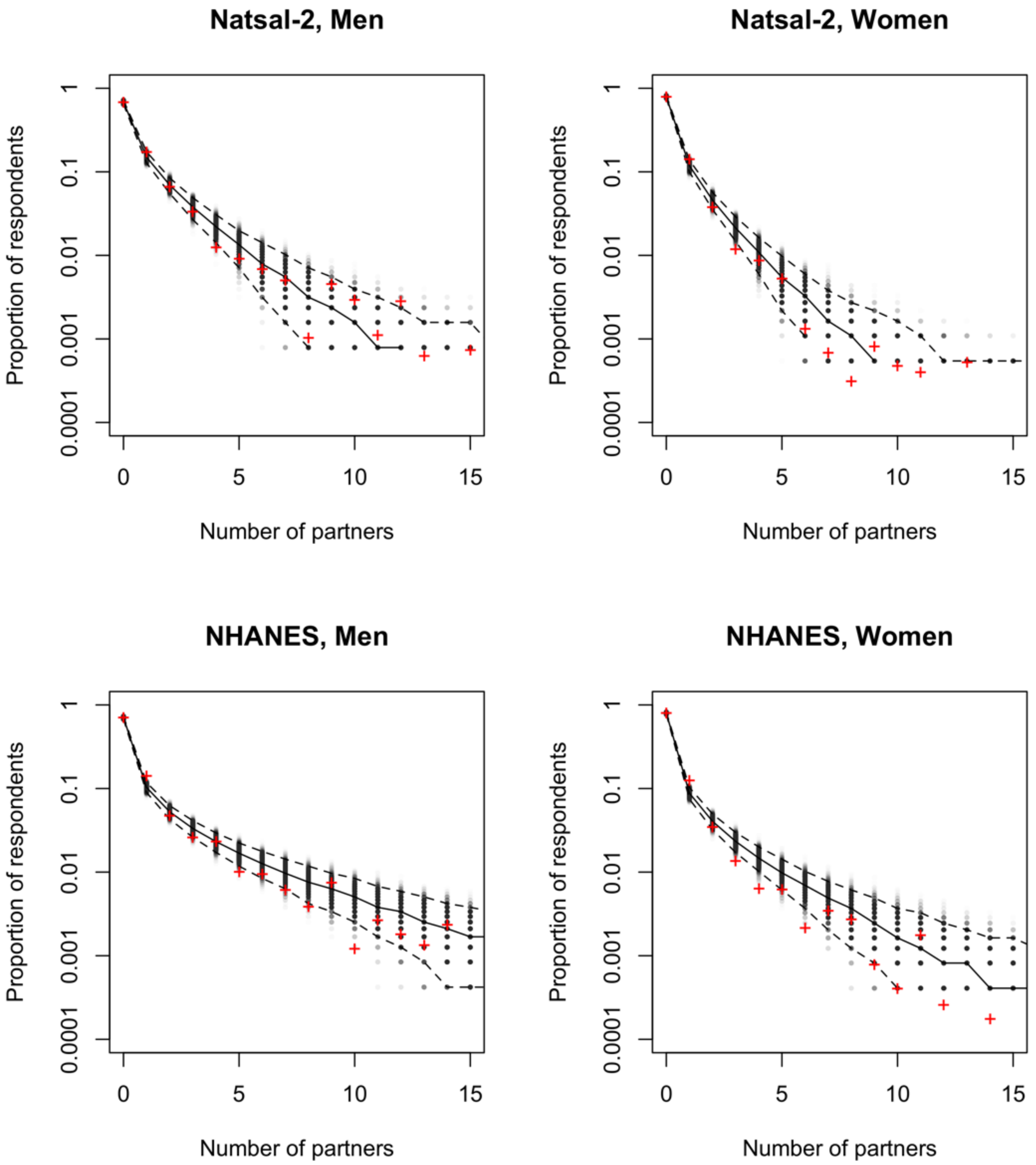


**Figure S5:** Simulated (grey) and observed (red) proportions of men (left) and women (right) reporting different numbers of new partners in the last year in the second National Study of Sexual Attitudes and Lifestyles (Natsal-2; top) and the National Health and Nutrition Examination Surveys (NHANES) 2009-2014 (bottom). In this model, the mean number of partnerships was not constrained to be equal between the sexes. Simulations are shown using transparent grey markers, so that several superimposed markers appear as a darker grey. The solid and dashed lines show the 2.5th, 50th and 97.5th centiles of the simulations. The observed data shown takes into account the survey weights.

### *ii. Condom use*

In Natsal-2 participants were asked, *With how many different women/men have you had vaginal (or anal) intercourse in the past year without using a condom?* To investigate the potential effects of condom use on our estimates, we used this question to estimate the number of new partners without a condom:

- If participants reported 0 partners without a condom then we classified them as having 0 new partners without a condom.
- If participants reported the same number of partners in the last year as partners without a condom (i.e. if all partners in the last year were without a condom) then we classified the number of new partners without a condom as the same as the total number of new partners.
- If neither of these conditions applied then we classified the number of new partners without a condom as the reported number of partners without a condom.

We used the same model as in the main analysis to estimate the transmission probabilities in partnerships where condoms were not always used. Figure S6 shows the posterior distributions compared to the posteriors in the main analysis.

As expected, the posterior distributions were shifted slightly to the right, suggesting higher transmission probabilities in partnerships without a condom, but the shift was small compared to the uncertainty in the estimates. The posterior median (95% credible interval) transmission probabilities were 40.1% (21.5-72.8)% from men to women and 31.6% (7.2-96.1)% from women to men. We conclude that it might be valuable for sexual behavior surveys to collect information on the annual number of new partnerships without a condom for parameter inference and predictive modelling. In the absence of such data, however, it is more reliable to calculate an average probability across all new partnerships, and we have no reason to suppose that such an average is not valid.

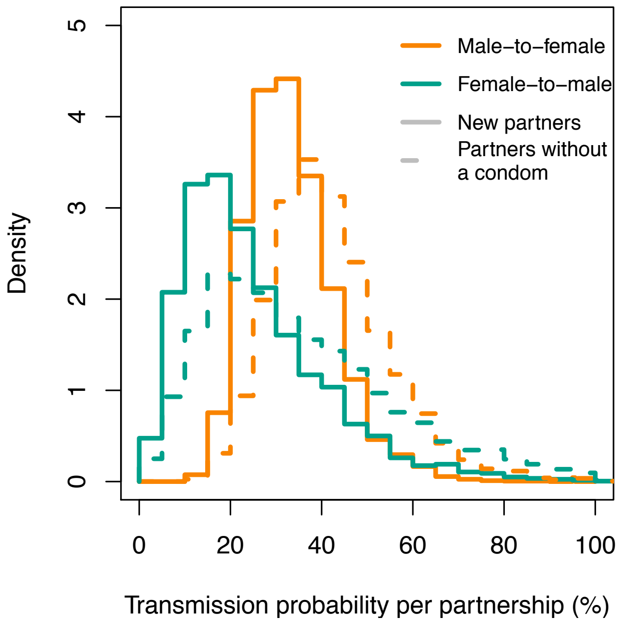


**Figure S6:** Posterior distributions for the per-partnership probability of chlamydia transmission, derived using data from the second National Study of Sexual Attitudes and Lifestyles (Natsal-2). The orange lines represent male-to-female transmission probability and the green lines female-to-male. The solid lines represent distributions inferred from reported numbers of new heterosexual partners, as in the main analysis. The dashed lines represent distributions inferred from the estimated number of new partners without a condom, as described in the text above.

### *iii. Assortative mixing*

The model reported in the main text assumes random mixing between men and women – that is, that for individual *j*, the probability that a partnership they form with a member of the opposite sex is a potential source of infection does not depend on *j*'s partnership formation rate. In fact, evidence indicates that sexual mixing is assortative,[12,13] although this is difficult to quantify precisely.

To investigate the potential effects of assortative mixing in our model, we reasoned that if individuals with more partners tend to form partnerships with others who also have more partners – and therefore the partners are more likely to be infected with chlamydia – then $\pi_p^{x'}$ would be higher in people with more partners. If the transmission probability were the same for every partnership then we would therefore expect the product

$$A_{x'\to x} = \pi_p^{x'} \rho_{x'\to x}$$

to be higher in people with more partners.

We ran an adapted model which allows $A$ to be different for men and women reporting different numbers of partners. If people with more partners are more likely to form partnerships with infected people then we would expect $A$ to be higher in those individuals.

Figure S7 shows the posterior distributions for $A$ that we inferred in men and women reporting different numbers of partners. For Natsal-2, although the posterior distributions for $A$ were slightly higher in people reporting no new partners, there was considerable overlap and therefore no evidence of significantly higher prevalence in partnerships presented to individuals with high partnership formation rate than to those with low formation rate. In NHANES the posterior distributions suggested higher values for $A$ in both men and women reporting no new partners: the opposite of what we would expect if there is assortative mixing. This pattern may arise if there is a higher transmission probability in slow-turnover partnerships, because they tend to last longer and have more sex acts during the infectious period, possibly with lower levels of condom use.

We found no evidence in either Natsal-2 or NHANES of higher $A$ in people reporting more partners, providing confidence that the random mixing in the model has not affected our results.

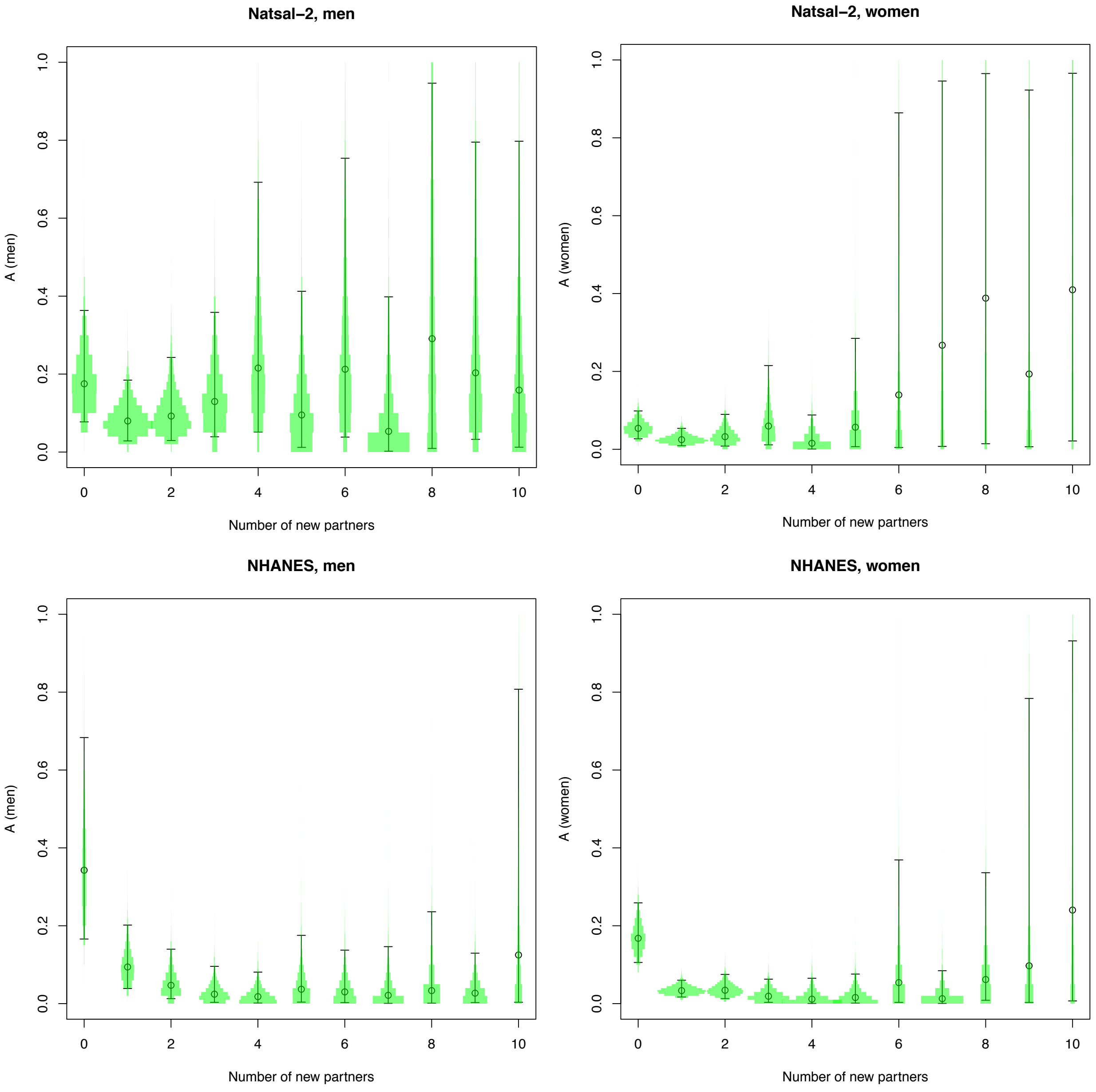


**Figure S7:** Posterior distributions for $A$ inferred separately for men and women reporting different numbers of partners. Error bars show median and 95% credible interval, and green polygons are histograms of the posteriors.

## 3. References


1. Price MJ, Ades AE, De Angelis D, et al., Mixture-of-exponentials models to explain heterogeneity in studies of the duration of Chlamydia trachomatis infection. *Stat Med.* 2013;32:1547-60.
2. Lewis J, Price MJ, Horner PJ, et al., Genital *C. trachomatis* infections clear more slowly in men than women, but are less likely to become established. *J Infect Dis.* 2017;216:237-244.
3. Johnson A, Fenton K, Copas A, et al., *National Survey of Sexual Attitudes and Lifestyles, 2000-2001* [computer file]. Colchester, Essex: UK Data Archive [distributor], 2005. SN: 5223, http://doi.org/10.5255/UKDA-SN-5223-1.
4. Centers for Disease Control and Prevention (CDC)} and National Center for Health Statistics (NCHS). *National Health and Nutrition Examination Survey Data.* [computer file]. Hyattsville, MD: U.S. Department of Health and Human Services, Centers for Disease Control and Prevention [distributor].
5. Handcock MS and Jones JH, Likelihood-based inference for stochastic models of sexual network formation *Theor Popul Biol.* 2004;65:413-422.
6. Gelman A, Carlin JB, Stern HS, et al.. Bayesian Data Analysis. Chapman &Hall/CRC Press, London, third edition. 2013.
7. Geisler WM, Wang C, Morrison SG, et al., The natural history of untreated *Chlamydia trachomatis* infection in the interval between screening and returning for treatment. *Sex Transm Dis.* 2008;35:119-123.
8. Stan Development Team. RStan: the R interface to Stan, Version 2.10.1. http://mc-stan.org/interfaces/rstan.html Accessed 7 June 2016.
9. R Core Team. R: A Language and Environment for Statistical Computing. 2016.
10. Chandra N, Soldan K, Dangerfield C, et al., Filling in the gaps: estimating numbers of chlamydia tests and diagnoses by age group and sex before and during the implementation of the English National Screening Programme, 2000 to 2012. *Eurosurveillance.* 2017;22:30453.
11. Centers for Disease Control and Prevention. *NCHHSTP AtlasPlus*. https://www.cdc.gov/nchhstp/atlas/index.htm. Accessed 3 December 2019.
12. Aral SO, Hughes JP, Stoner B, et al., Sexual Mixing Patterns in the Spread of Gonococcal and Chlamydial Infections *Am J Public Health.* 1999;89:825-833.
13. Prah P, Copas AJ, Mercer CH, et al., Patterns of sexual mixing with respect to social, health and sexual characteristics among heterosexual couples in England: analyses of probability sample survey data *Epidemiol Infect.* 2015;143:1500-1510.
